# The Relationship between Tumor-infiltrating Lymphocytes (TILs) and Nasopharyngeal Carcinoma (NPC): A Systematic Review

**DOI:** 10.22038/ijorl.2021.51405.2733

**Published:** 2021-07

**Authors:** Awal Prasetyo, Jethro Budiman, Udadi Sadhana

**Affiliations:** 1 *Department of Biomedical Science, * *Faculty of Medicine, * *Diponegoro University, Semarang, Indonesia.*; 2 *Emergency Unit of Panti Wilasa Citarum Hospital, * *Semarang, Indonesia* *.*; 3 *Department of * *Anatomic Pathology,* * Faculty of Medicine, Diponegoro University - dr. * *Kariadi Hospital, * *Semarang, Indonesia* *.*

**Keywords:** Immunology, Nasopharyngeal Carcinoma, Tumor-Infiltrating Lymphocytes

## Abstract

**Introduction:**

Nasopharyngeal carcinoma (NPC) is a rare and aggressive head and neck squamous cell carcinoma worldwide. Tumor-infiltrating lymphocytes (TILs) have been studied and reported to be effective targets of drugs on cancer and were related to the prognostic value. The aim of the study was to look systematically into the current literature and carefully analyze the results to explore the relationship of TILs and NPC.

**Materials and Methods:**

Three independent reviewers conducted the literature search, searching for articles published in January 2000-January 2020 and fulfilling inclusion and exclusion criteria. The lead author independently assessed the risk of bias of each of the included studies and discussed their assessments with the other two authors to achieve consensus. Of the 1233 articles identified in database searching, 12 articles met the criteria for this review.

**Results:**

The majority of the study designs were cohort (9 of 12 studies). Most of the studies discussed the prognostic significance of TILs in NPC (nine studies), two studies reported the expanded TILs for the treatment of NPC, and one study reported TILs based on one gene expression.

**Conclusion:**

TILs in NPC are related to the prognostic factor and development of the immunotherapy. High TILs were associated with better outcome and survival rate; and TILs have been claimed to reflect an effective anti-tumor immune response, immune response inducer, delayed tumor progression, and improving the cancer-immune microenvironment. The understanding of TILs in NPC based on gene expression becomes important information to learn more about the relationship of TILs and NPC.

## Introduction

 Nasopharyngeal carcinoma (NPC) is a rare and aggressive head and neck squamous cell carcinoma worldwide; but it is endemic in a few areas, like Southern China, Southeast Asia, North Africa, and the Arctic (1-4). 

The incidence in Southern China and Southeast Asia is high with the majority of tumors being undifferentiated and non-keratinizing carcinomas. On the contrary, NPCs of non-endemic areas (such as Northern Europe) can be keratinizing or non-keratinizing (5). Based on the Global Cancer Observatory (GLOBOCAN), 129.079 new cases of NPC were diagnosed in 2018 and were associated with 72.987 deaths (5,6). 

One of the most striking and consistent characteristics of NPC is the presence of a very abundant lymphocyte infiltrate with a high rate of local invasion and locoregional lymphatic metastasis (5,7). 

The tumor has a multifactorial etiology and differs from other head and neck cancers by characteristic histological findings (3,8). The prognosis of NPC was improved signiﬁcantly with the development of the treatment (radiotherapy, chemotherapy, and surgical) (1,3). For recent years, the prognosis and the strategy treatment of NPC used the tumor–node–metastasis (TNM) cancer staging system, but it remained large variations in the patient's prognosis who were also undergoing the similar treatment with the same stage so the TNM system was maybe not enough to evaluate the entire NPC status or guide treatments (1,9).

The malignant phenotypes of cancers are also deﬁned by the immune cells activated in the tumor microenvironment (TME) (2,10). TME consists of immune cells (including tumor-infiltrating lymphocytes/TILs), endothelial cells, mesenchymal cells, inﬂammatory mediators and extracellular matrix molecules (1,2,11). 

In TME, tumor-infiltrating lymphocytes (TILs) are one major type of nontumor components and have been validated for diagnostic and prognostic assessment of tumors (1,2,11). 

TILs are immune cells that are triggered by the host's immune response to the tumor; including T cells (CD4+ T helper lymphocytes/ Th, CD8+ cytotoxic T lymphocytes/ CTLs, and FOXP3+ regulatory T-cells/ Tregs), macrophages, dendritic cells, and mast cells (1,12,13). TILs have been studied and reported to be effective targets of drugs on cancer and were related to the prognostic value. Mechanism studies conﬁrmed that TILs have a dual role by conducting both host immune defense and tumor progression (1,5). The relationship of TILs and NPC have been reported and explained in various studies, but there was no systematic review about the relationship of them. 

The aim of the study was to look systematically into the current literature and carefully analyze the results to explore the relationship of TILs and NPC.

## Materials and Methods

**Scope of the review: inclusion and exclusion criteria**

**Inclusion criteria:**

1. Publication type:

- full-text articles discussing the relationship of TILs and NPC
- primary studies of every design (case study, case series, cross-sectional, case control, cohort, and clinical trial)

2. Languange of publication: english

3. Time of publication: January 2000-January 2020 

4. Methodology: studies included must explain the relationship of TILs and NPC

**Exclusion criteria:**

1. Objective and outcome measures are not relevant (are not about the relationship of TILs and NPC)

2. Confounding variables are related to outcome in the relationship of TILs and NPC

**Literature search**

This systematic review was conducted in accordance with Cochrane handbook for systematic reviews and is reported by using the guideline of preferred reporting items for systematic review and meta-analysis (PRISMA) (14,15). A systematic search strategy was followed in these electronic databases: Cambridge Core, Clinical Key, Ebsco, Emerald Insight, JSTOR, Medline, Nature, Proquest, Pubmed, Science Direct, Scopus, and Springer Link.

The search was conducted using the following keywords for title and abstract: (tumor infiltrating lymphocyte) AND (nasopharyngeal cancer OR nasopharyngeal carcinoma OR nasopharyngeal tumor). 

The reference lists of retrieved papers were also examined to avoid missing any published data (including grey literature in the library and hand searching). 

**Data collection and analysis**

Studies were selected for retrieval after two independent reviewers (AP and JB) had collected titles and abstracts identified in electronic searches. 

The results of the two reviewers were compared by a third independent reviewer (US), and any differences of opinion were resolved by discussion. Full papers from potential studies were independently assessed by the investigators (AP and JB).

All studies selected for this systematic review were screened by two reviewers independently to validate the results (AP and JB). The data from all retrieved studies were presented in a summary table featuring key points of each study (Table.5). The following data were collected: first author, country, and year; study design, sample size and characteristic, outcome measure, and result.

**Quality assessment**

The lead author independently assessed the risk of bias of each of the included studies and discussed their assessments with the other two authors to achieve consensus.

Newcastle-ottawa scale adapted for cross-sectional studies, newcastle–ottawa scale cohort version, cochrane risk of bias were used to assess the methodological quality of the studies (14,16–18). 

Newcastle-ottawa scale adapted for cross-sectional studies was used to assess cross-sectional studies, interpretation of total score was: 9 to 10 points were considered in very good studies, 7 to 8 points were considered in good studies, 5 to 6 points were considered in satisfactory studies, and 0 to 4 points were considered in unsatisfactory studies (16). Newcastle–ottawa scale cohort version was used to assess cohort study, interpretation of total score was: ≥7 points were considered in good studies, 5-6 points were considered in fair studies, <5 points were considered in poor studies (17–20).

Cochrane risk of bias was used to asses randomized control trial study (experimental), whose results were either high risk or some concerns or low risk (14).

## Results

**Selection of articles for review**

**Fig 1:**
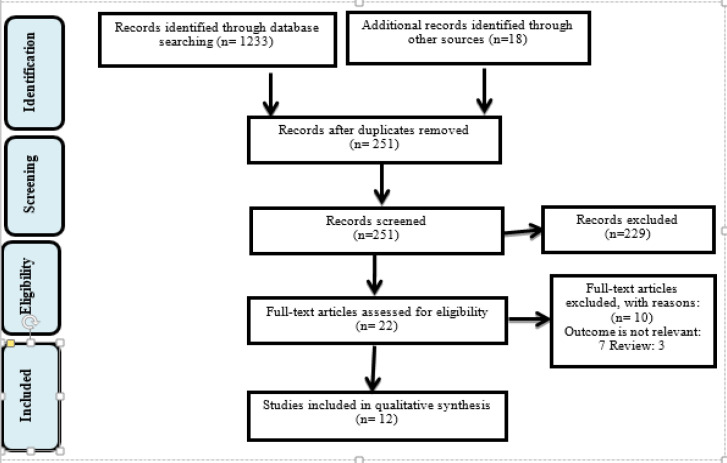
Preferred reporting items for systematic reviews and meta-analysis (PRISMA)

Figure 1 summarized the identiﬁed, screened, and included articles for review. Initially, 1233 peer-reviewed articles were identiﬁed from electronic databases and an additional 18 articles were identiﬁed through other sources (including search engine, gray paper, and hand searching). 

After removing duplicates, 251 articles remained for the title and abstract screening. Articles that did not meet the inclusion and exclusion criteria were not further screened. Twenty-two articles were screened for eligibility of which 12 articles met all the inclusion criteria.

**Assessment of study validity (risk of bias)**

All eligible studies were associated with the relationship of TILs and NPC. 

Table 1 provides quality scores for cross-sectional study and got 5 points that were considered in the satisfactory study. Table 2 provides quality scores for cohort studies, all studies get 5-7 points that were considered in fair and good studies. 

Table 3 and table 4 provide quality scores for randomized control trial study, all studies were considered in some concerns and low risk. 

**Table 1:** Newcastle-Ottawa scale adapted for cross-sectional study

No.	First author, year	Selection				Comparability	Outcome		Total
		1	2	3	4		1	2	
1.	Luo M, 2019 (1)				**		**	*	5

**Table 2:** Newcastle-Ottawa scale (cohort study)

No.	First author, year	Selection				Comparability	Outcome			Total
		1	2	3	4		1	2	3	
1.	Almangush A, 2018^5^			*	*		*	*	*	5
2.	Larbcharoensub N, 2018^21^			*	*		*	*	*	5
3.	Lu J, 2018^7^			*	*		*	*	*	5
4.	Ono T, 2018^2^			*	*		*	*	*	5
5.	Wang Y, 2018^9^	*	*	*	*		*	*	*	7
6.	Chan OSH, 2017^22^			*	*		*	*	*	5
7.	Ooft ML, 2017^23^			*	*		*	*	*	5
8.	Zhang Y, 2010^24^			*	*		*	*	*	5
9.	Oudejans J J, 2002^25^			*	*		*	*	*	5

*Maximum point for comparability were 2

**Table 3:** Cochrane risk of bias 1: Li J, 2015^26^

**No.**	**Domain**	**Description of domain**	**Results**
1.	**Domain 1**	**risk of bias arising from the randomization process**	**some concerns**
2.	Domain 2	risk of bias due to deviations from the intended interventions (effect of adhering to intervention)	low risk
3.	Domain 3	missing outcome data	low risk
4.	Domain 4	risk of bias in measurement of the outcome	low risk
5.	Domain 5	risk of bias in selection of the reported result	low risk
			

**Table 4:** Cochrane risk of bias 2: He J, 2012^27^

**No.**	**Domain**	**Description of domain**	**Results**
1.	Domain 1	**risk of bias arising from the randomization process**	**some concerns**
2.	Domain 2	risk of bias due to deviations from the intended interventions (effect of adhering to intervention)	low risk
3.	Domain 3	missing outcome data	low risk
4.	Domain 4	risk of bias in measurement of the outcome	low risk
5.	Domain 5	risk of bias in selection of the reported result	low risk
			

**Study characteristic**

Study characteristics for the included studies could be seen in table 5. The majority of the study designs were cohort (9 of 12). Most of the studies discussed the prognostic significance of TILs in NPC (nine studies), two studies reported the expanded TILs for the treatment of NPC, one study reported TILs based on one gene expression.

**Table 5:** Study characteristic

**No.**	**First author, country, year**	**Study design**	**Sample size (n) and characteristic:age (year), gender (male, female), NPC characteristic**	**Outcome measure**	**Result**
1.	Almangush A, Finland, 2018^5^	Cohort	115 Characteristic Age: 58 (12-85) Gender: 80,35 NPC Characteristic: Type I: 28 Type II: 19 Type III: 68 Stage I: 15 Stage II: 29 Stage III: 40 Stage IV: 31	The prognostic significance of TILs in NPC	NPC with low intra-tumoral TILs had poor OS (HR: 2.55, 95%CI: 1.60-4.50, P<0.001) and poor DSS (HR: 2.02, 95%CI: 1.16-3.52, p: 0.015). Keratinized tumors with low intra-tumoral TILs were associated with a poor OS (HR: 3.94, 95%CI: 2.17-7.15, P< 0.001) and a poor DSS (HR:2.97, 95%CI: 1.46-6.05, p: 0.009).
2.	Chan OSH, Hongkong, 2017^22^	Cohort	161 Characteristic Age: 53 (27-88) Gender: 117,44 NPC Characteristic: Stage I: 2 Stage II: 13 Stage III: 78 Stage IV: 68	Characterization of PD-L1 expression and immune cell inﬁltration in nasopharyngeal cancer (the prognostic significance of TILS in NPC)	75% of tumors expressed PD-L1 on TILs and 24% on TC. High CD8+ TILs were associated with better OS (HR: 0.53, 95%CI: 0.34-0.84, P: 0.0059). and PFS (HR: 0.57, 95%CI: 0.38-0.85, p: 0.006).
3.	He J, China, 2012^27^	Experimental	15 Characteristic: not described	Ex vivo expansion of TILs from NPC for adoptive immunotherapy	Young TIL cultures comprised of more than 90% of CD3+ T cells, a variable percentage of CD3+CD8+ and CD3+ CD4+ T cells, and less than 10% of CD3-CD16+ NK cells, a similar phenotype of EBV-CTL cultures from PBMCs. TIL cultures secreted high levels of the Th1 cytokines, IFNγ and TNF-α, and low levels of the Th2 cytokines, IL-4 and IL-10. Young TILs could recognize autologous EBV-transformed B lymphoblast cell lines, but not autologous EBV-negative blast cells or allogeneic EBV-negative tumor cells.
4.	Larbcharoensub N, Thailand, 2018^21^	Cohort	114 Age: 51.6±12.7 Gender: 77, 37 NPC Characteristic: Stage I: 3 Stage II: 14 Stage III: 41 Stage IV: 48	Characterization of PD-L1 and PD-1 expression and CD8+ TILs in Epstein-Barr Virus-associated NPC (the prognostic significance of TILS in NPC)	PD-L1 was expressed in ≥ 1% of TCs in 69% of patients, in ≥ 50% of TCs in 12% of patients, and ≥ 5% of either TCs or inﬁltrating immune cells in 71% of patients. CD8+ TILs were present in tumors from all patients. High CD8+ TILs levels in NPC were associated with a longer OS.
5.	Li J, China, 2015^26^	Experimental	23 Characteristic Age: 45.5 (29-62) Gender: 18, 5 NPC Characteristic: Type III at an advanced stage (stage III-IV) without distant organ metastasis at diagnosis	The safety and antitumor activity of ACT using expanded TILs following CCRT in patients with locoregionally advanced NPC	Three patients failed to produce sufficient TILs (drop out). Only mild AEs including grade 3 neutropenia (1/23, 5%) consistent with immune-related causes, were observed. 19 of 20 patients exhibited an objective antitumor response, and 18 patients displayed DFS longer than 12 mo after ACT.
6.	Lu J, China, 2018^7^	Cohort	197 Characteristic Age: not described Gender: 146,51 NPC Characteristic: Stage I: 10 Stage II: 30 Stage III: 62 Stage IV: 95	Analysis of inflammatory cell infiltration and the prognostic impact on NPC	The patients with NPC with a low density of FOXP3+, CD8+ TILs, neutrophils, and mast cells showed a significantly longer OS and PFS (p: 0.006, p: 0.018, p: 0.002). The prognostic impacts of CD8+ TILs (HR: 1.700, p: 0.036) and FOXP3+ (HR:1.714, p: 0.034) on OS were not proved.
7.	Luo M, China, 2019^1^	Cross sectional	NPC: 12 Normal: 4 Characteristic: not described	TILs in NPC based on gene expression	NPC samples contained a higher proportion for M1 macrophages, whereas memory B cells and CD4 memory resting T cells were relatively lower. High M1 macrophages, memory B cells, and CD4 memory resting T cells were associated with a better survival rate (p: 0.286, p: 0.509, p: 0.048).
8.	Oudejans J J, Indonesia, 2002^25^	Cohort	43 Characteristic Age: 48 Gender: 32, 11 NPC Characteristic: without evidence of distant metastases	The prognostic significance of GrB/ CD8+ TILs in NPC	The presence of a high percentage (>25%) of GrB + TILs appeared to be a very strong predictor of a rapid fatal clinical outcome, independent of stage.
9.	Ono T, Japan, 2018^2^	Cohort	66 Characteristic Age: 59.5 (13-85) Gender: 54,12 NPC Characteristic: Type I: 18 Type II-III: 48 Stage I: 3 Stage II: 18 Stage III: 25 Stage IV: 20	The prognostic significance of TILs in NPC	CD8+ TILs were significant predictive factor for PFS (HR: 0.36, 95%CI: 0.15-0.88, p: 0.025) and OS (HR: 0.30, 95%CI: 0.12-0.73, p: 0.008).
10.	Ooft ML, Netherland, 2017^23^	Cohort	92 Characteristic Age: 53.45 Gender: 63,26 NPC Characteristic: Type I: 12 Type II-III: 76	The prognostic significance of TILs in NPC	EBV positive NPC contains signiﬁcantly more CD3+, CD4+, and CD8+ TILs than EBV negative NPC. In the whole NPC group, increased CD8+ count is associated with better OS (HR: 0.219. 95%CI: 0.075-0.640), but also in cases with PDL1 co-expression (HR: 0.073, 95%CI: 0.010-0.556). In EBV positive NPC co-expression of CD8+ and PDL-1 showed better DFS (HR:0.407, 95%CI: 0.195-0.850) and OS (HR 0.170, 95%CI: 0.037–0.787).
11.	Wang Y, China, 2018^9^	Cohort	Training set: 591 Gender: 363, 228 NPC Characteristic: Stage I-II: 127 Stage III-IV: 464 Validation set: 584 Gender: 330, 254 NPC Characteristic: Stage I-II: 130 Stage III-IV: 454 Independent set: 304 Gender: 218, 86 NPC Characteristic: Stage I-II: 44 Stage III-IV: 260	The prognostic significance of TILs in NPC	High TILs in the training set were significantly associated with favorable DFS (HR: 0.41, 95%CI: 0.28-0.58, p< 0.001), OS (HR 0.42, 95%CI: 0.27-0.64, p < 0.001), DMFS, (HR: 0.37, 95%CI: 0.23-0.58, p< 0.001 and LRRFS (HR: 0.43, 95%CI :0.25-0.73, p: 0.002).
12.	Zhang Y, China, 2010^24^	Cohort	106 Characteristic Age: 49 (22-73) Gender: 84,22 NPC Characteristic: Type I: 1 Type II: 13 Type III: 92 Stage I-II: 38 Stage III-IV: 68	The prognostic significance of TILs in NPC	The density of FOXP3+ TILs or Foxp3+ TILs combined with GrB+ TILs together were associated with better OS and PFS (p< 0.01). Low density of CD8+TILs or high ratio of FOXP3+TILs to CD8+TILs was correlated with better PFS in early-stage patients (Stages I and II, p< 0.05).

**Abbreviation:** ACT: adoptive cell therapy AEs: adverse events          CCRT: concurrent chemoradiotherapy           CD: cluster of differentiation            CI: confidence interval            DFS: disease-free survival           DMFS: distant metastasis-free survival           DSS: disease specific survival            EBV: epstein-barr virus            FOXP3: forkhead box p3 GrB: Granzyme B           HR: hazard ratio           IFN-γ: interferon-gamma            IL: interleukin            LRRFS: local-regional recurrent free survival

mo: month NK: natural killer            NP: non-malignant nasopharyngeal            OS: overall survival            p: probability            PBMC: peripheral blood mononuclear cells            PFS: progression-free survival            T_reg_: regulatory T cells            TCs: tumor cells

## Discussion

**The relationship of TILs and NPC**

The prognostic significance of TILs in NPC was explained in nine studies. Six studies showed that high TILs were associated with better outcomes and survival rates. Wang Y, et al. (2018) reported that stromal TILs were found to be a superior parameter with a high reproducibility than intratumoral TILs. The limited number and restricted range of intratumoral TILs might be an explanation for the inferior prognostic value of intratumoral TILs; intratumoral TILs are more heterogeneous and are relatively difﬁcult to observe on hematoxylin-eosin stained slides. As the combination of intratumoral and stromal TILs, TILs seem to be the strongest survival predictor of NPC outcomes (9). Generally, the predominance of TILs has been claimed to reflect an effective anti-tumor immune response, immune response inducer, delayed tumor progression, and improving the cancer-immune microenvironment (2,5,9,23,26). TILs also can recognize tumor antigens and instigate tumor rejection (23) Treg cells in TILs can suppress naïve T cells and effector T cells and are defined as immune suppression cells that inhibit antitumor immunity and help tumor cell immune evasion. The mechanisms of suppressing proliferation of naïve or effector T cells of these Treg cells are controlled by cell-to-cell contact or secreting cytokines interleukin-10 (IL-10) and tumor growth factor-beta (TGF-β). However, it has been identified that some tumor-derived Treg cells with the tumor antigen specificity could recognize the autologous antigen-specific tumor cell and secret interferon-gamma (IFN-γ) *in vitro (*5,24).*O*n the contrary, we found three studies that explain the correlation of high TILs and poor prognosis in NPC. An explanation might be the functional inactivation of CD8^+^ CTL (cluster of differentiation 8-cytotoxic T lymphocyte) in NPC (7). Li J, et al. (2007) has demonstrated that CD8^+ ^CTLs could increase PD-1 (program death-1) expression and reduce CD3 expression, resulting in an impaired tumor-specific immunity (4). 

In addition, CD8^+^ CTL may upregulate the expression of PD-L1 (program death ligand-1) and indoleamine-2,3-dioxygenase in tumor cells, recruit Tregs in the tumor microenvironment, and then promote tumor immune escape by the production of CCL22 (C-C motif chemokine ligand 22) and IFN-γ (7). Lu J, et al. (2018) reported that CTL has been assigned an important role in antitumor immunity; but in the research, CD8^+ ^CTL density was positively associated with poor PFS in early-stage patients. Therefore, a possible explanation for this result is that a CD8^+ ^CTL function is impaired thus the CD8^+ ^CTL could not kill the tumor cell in NPC TILs (7). Another possibility is that the acute immune response could induce the spreading of tumor cells to the regional lymphoid node (24). These three studies seem to contradict the most finding, but the discrepancy may be due to small sample sizes, racial origins, and limited statistical power (9,24).

Two studies explained the use of TILs in immunotherapy (26,27). Adoptive cell therapy (ACT) using TILs have an antitumor effect and can induce an immune response in NPC. This study demonstrated that NPC patients can tolerate adoptive cell therapy with TILs following CCRT (concurrent chemoradio- therapy) and experience sustained antitumor activity and anti-EBV (epstein-barr virus) immune responses. Additionally, a larger phase II trial is in progress. Although TILs have shown some therapeutic effects, their prognostic effect has been overlooked. The study conducted by Li J, et al. (2015) emphasized that TILs are a powerful, independent predictor of DFS (disease-free survival) in NPC patients and deserve further investigation (9,26). Another study explained about ex vivo expansion of TILs from NPC patients for adoptive immunotherapy. Ex vivo expansion of TILs from NPC patients may have several potential advantages. First, the experimental method for establishing young TILs is simple and rapid, and TIL cultures can be successfully established for most NPC patients with tumor biopsy tissues. Second, the young TILs cultures have a low non-specific activity to human leukocyte antigen (HLA) mismatched cells because these cultures contain a high percentage of CD3^+^ T cells and a low percentage of CD3-CD16^+^ NK cells so can minimize the rejection reaction. Finally, young TIL cultures have a stable EBV specific activity and contain a higher percentage of tumor recognized EBV antigen-specific T cells compared to EBV-CTLs stimulated by auto LCLs (lymphoblast cell lines) from peripheral blood (26,27). One study explained about TILs in NPC based on gene expression by using CIBERSORT (a gene expression-based deconvolution algorithm)(1). The fraction of 22 immune cells in NPC was associated with tumorigenesis (gain of malignant properties in normal cells, which have been generalized as the hallmarks of cancer), which is potentially useful for the development of immunotherapy (1,28). This research found that NPC samples contained a higher proportion for M1 macrophages, whereas memory B cells and CD4 memory resting T cells were relatively lower; and all of this may be associated with tumorigenesis of NPC. These differences of immune cells might be important determinants for the prognosis. The survival analyses based on the cancer genome atlas (TCGA) database showed that CD4 memory resting T cells would be a predictive outcome signature in NPC. Therefore, M1 macrophages, memory B cells, and CD4 memory resting T cells may play pivotal roles in the development of NPC and the differentiation may be possible therapeutic targets (1,5,12,13).

**Strength and limitation of the study**

The present systematic review involved studies that reported 12 studies related to the relationship of TILs and NPC (nine studies about the prognostic significance of TILs in NPC, two studies about the development treatment of TILs in NPC, and one study about gene expression of TILs in NPC). In addition, a comprehensive literature search was followed, as well as bias protection methods such as three independent reviewers. The limitation of the study was related to the minimal sample and one area of each study, most of the studies were observational studies (there were only two randomized controlled trial study/RCT), and there was language bias (only english language was included in this study). 

**Future implication**

The current systematic review is expected to be a scientific consideration to clinician-related the application of TILs in NPC, like as prognostic factor or the development of the treatment of NPC, and general information related to the relationship of TILs and NPC for the public society. Further research is needed on the other relationship of TILs and NPC.

## Conclusion

Tumor-infiltrating lymphocytes (TILs) in nasopharyngeal carcinoma (NPC) are related to the prognostic factor and development of the immunotherapy. High TILs were associated with better outcome and survival rate; and TILs have been claimed to reflect an effective anti-tumor immune response, immune response inducer, delayed tumor progression, and improving the cancer-immune micro- environment. The understanding of TILs in NPC based on gene expression becomes important information to learn more about the relationship of TILs and NPC. 
